# DSIF and RNA Polymerase II CTD Phosphorylation Coordinate the Recruitment of Rpd3S to Actively Transcribed Genes

**DOI:** 10.1371/journal.pgen.1001173

**Published:** 2010-10-28

**Authors:** Simon Drouin, Louise Laramée, Pierre-Étienne Jacques, Audrey Forest, Maxime Bergeron, François Robert

**Affiliations:** 1 Institut de Recherches Cliniques de Montréal, Montréal, Québec, Canada; 2 Département de Médecine, Faculté de Médecine, Université de Montréal, Montréal, Québec, Canada; The University of North Carolina at Chapel Hill, United States of America

## Abstract

Histone deacetylase Rpd3 is part of two distinct complexes: the large (Rpd3L) and small (Rpd3S) complexes. While Rpd3L targets specific promoters for gene repression, Rpd3S is recruited to ORFs to deacetylate histones in the wake of RNA polymerase II, to prevent cryptic initiation within genes. Methylation of histone H3 at lysine 36 by the Set2 methyltransferase is thought to mediate the recruitment of Rpd3S. Here, we confirm by ChIP–Chip that Rpd3S binds active ORFs. Surprisingly, however, Rpd3S is not recruited to all active genes, and its recruitment is Set2-independent. However, Rpd3S complexes recruited in the absence of H3K36 methylation appear to be inactive. Finally, we present evidence implicating the yeast DSIF complex (Spt4/5) and RNA polymerase II phosphorylation by Kin28 and Ctk1 in the recruitment of Rpd3S to active genes. Taken together, our data support a model where Set2-dependent histone H3 methylation is required for the activation of Rpd3S following its recruitment to the RNA polymerase II C-terminal domain.

## Author Summary

Acetylation of histone N-terminal tails occurs on nucleosomes as a gene is being transcribed, therefore helping the RNA polymerase II traveling through nucleosomes. Histone acetylation, however, has to be reversed in the wake of the polymerase in order to prevent transcription from initiating at the wrong place. Rpd3S is a histone deacetylase complex recruited to transcribed genes to fulfill this function. The Rpd3S complex contains a chromodomain that is thought to be responsible for the association of Rpd3S with genes since it interacts with methylated histones, a feature found on transcribed genes. Here, we show that the recruitment of Rpd3S to transcribed genes does not require histone methylation. We found that Rpd3S is actually recruited by a mechanism implicating the phosphorylation of the RNA polymerase II C-terminal domain and that this mechanism is regulated by a transcriptional elongation complex called DSIF. We propose that the interaction between the Rpd3S chromodomain and methylated histones helps anchoring the deacetylase to its substrate only after it has been recruited to the elongating RNA polymerase.

## Introduction

Histone acetylation was the first covalent histone modification shown to be involved in transcription regulation. Indeed, histones at the promoter of active genes tend to be hyper-acetylated while repressed genes have promoters with hypo-acetylated nucleosomes. It is now well established that this is due to the recruitment of histone acetyltransferases (HATs) and histone deacetylases (HDACs) by transcriptional activators and repressors, respectively [1]. The first described and best characterized HDAC is Rpd3. The repressive effect of yeast Rpd3 on transcription has been well studied over the last 15 years, paving the way for the characterization of its mammalian orthologs [2]. Yeast Rpd3 is recruited to the promoters of specific genes by DNA-binding repressors, leading to the repression of many important pathways such as stress response, meiosis, the cell cycle and others [3]–[19]. Moreover, Rpd3 also plays roles in silencing [20]–[27], DNA replication [28]–[31] and recombination [32], [33].

Recent proteomic studies have determined that Rpd3 is found in two distinct complexes: the large (Rpd3L) and the small (Rpd3S) complex [34], [35]. Both complexes share a core composed of Rpd3, Sin3 and Ume1. The large complex is composed of 11 additional proteins whereas the small complex contains only two additional subunits, namely Rco1 and Eaf3. While Rpd3L is likely responsible for the repressive function of Rpd3, the function of Rpd3S remains far less understood. Recent work by several groups has shown that Rpd3S is involved in the suppression of cryptic transcription [34], [36], [37] and that its activity is linked to the Set2 histone methyltransferase (HMT). Furthermore, *in vitro* studies have shown that Rpd3S is recruited to H3K36-methylated nucleosomes and that its Rco1 and Eaf3 subunits are essential for this recruitment [34], [35], [37], [38]. Rco1 mediates interactions with histones in a modification-independent manner through a PHD zinc finger domain, while Eaf3 contains a methyl-lysine binding chromodomain (CHD) that is essential for recognition of H3K36-trimethylated (H3K36me3) nucleosomes *in vitro*. These studies also indicate that genome-wide histone acetylation levels on promoters and coding regions are altered when either H3K36 methylation or Rpd3S is disrupted.

Taken together, these data lead to a model where Rpd3S is recruited to coding regions through the interaction of Eaf3 with H3K36me3 in order to deacetylate nucleosomes after the passage of the transcriptional machinery [39]. Deacetylation would allow chromatin disrupted by elongating RNA polymerase II (RNAPII) to return to a more ordered and compact structure, thereby restoring an environment hostile to cryptic transcription initiation within the coding region. However, this model relies heavily on the *in vitro* observation that the Eaf3 CHD binds preferentially to H3K36me3 peptides or nucleosomes [34], [35], [37], [38]. It was never formally demonstrated that this interaction is required for the targeting of Rpd3S to coding regions *in vivo*. In addition, whether Rpd3S is recruited to all transcribed genes or to only a subset of them has never been assessed. In order to address these questions, we performed genome-wide ChIP-chip experiments looking at both Rpd3S- and Rpd3L-specific subunits in wild type cells and in various mutants, including *set2*Δ and H3K36A (Table S1 and S2).

Quite interestingly, our data show that Rpd3S specifically binds to the coding region of actively transcribed genes whose promoters are also bound by Rpd3L. Surprisingly, the binding of Rpd3S to active genes is not dependant on Set2-mediated H3K36 methylation. However, methylation by Set2 is required for the activity of Rpd3S, as assayed by histone acetylation and RNAPII levels. We also provide *in vivo* evidence that Rpd3S is recruited to active genes via the phosphorylation of the RNAPII C-terminal domain (CTD). Finally, we show that the yeast DSIF transcription elongation factor negatively regulates Rpd3S recruitment. Based on these results, we propose that the recruitment and activity of Rpd3S on ORFs depend on a two steps mechanism: an initial recruitment to the elongation complex -coordinated by DSIF and RNAPII phosphorylation-, followed by the H3K36me3-dependant modulation of Rpd3S activity through the Eaf3 chromodomain.

## Results

### Rpd3S is not recruited to all transcribed genes

**Figure 1:**
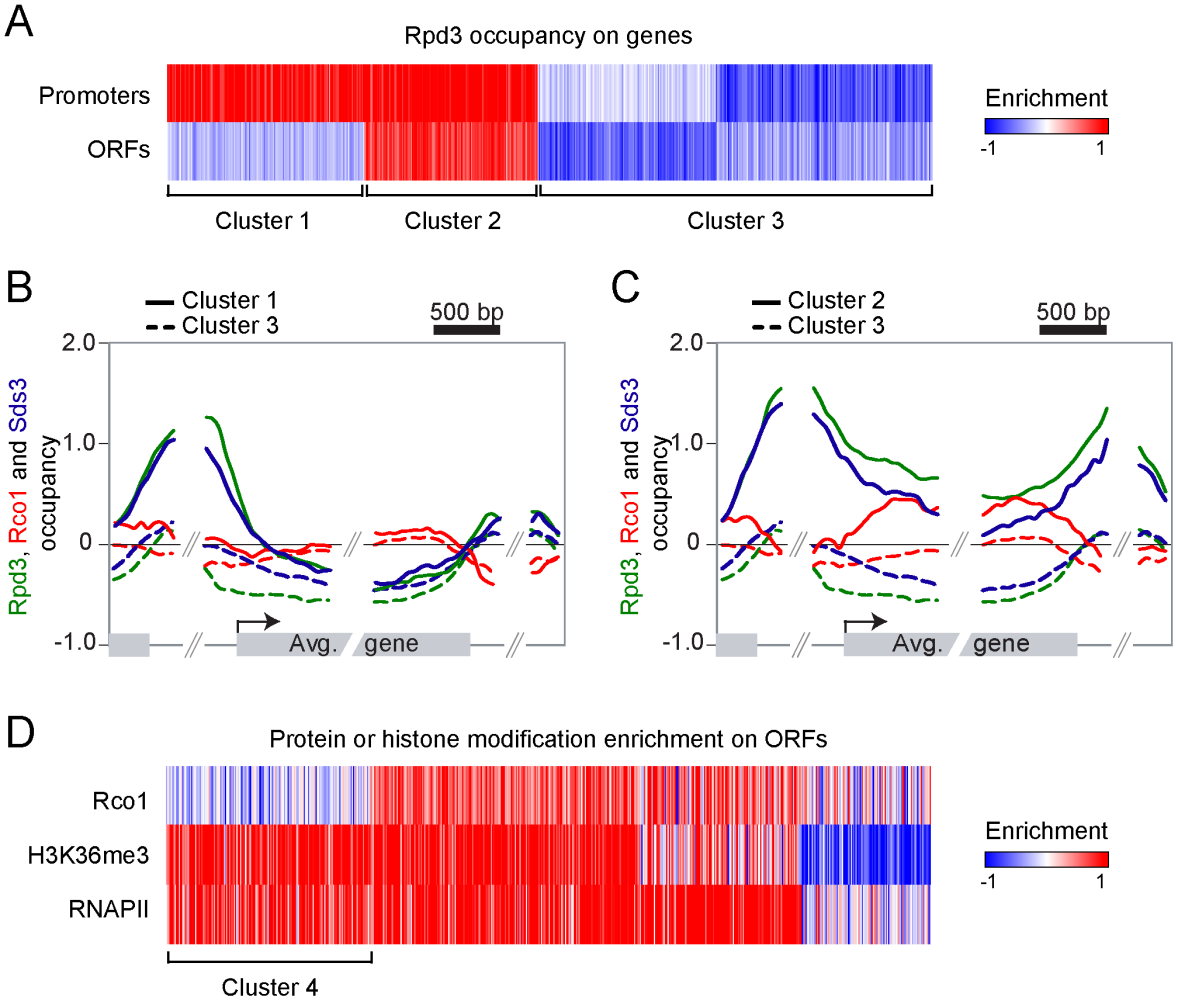
Rpd3S and Rpd3L target overlapping sets of genes. (A) Self-organizing map (SOM) clustering of Rpd3 binding on promoter and ORFs of the 3693 genes with no missing value. Red indicates enriched (bound) regions and blue represents depleted regions. (B,C) Mapping of Rpd3 (green), Rco1 (red) and Sds3 (blue) on the 954 genes from cluster 1 (solid line, genes where Rpd3 is present both on ORFs and promoters) and on the 1906 genes from cluster 3 (dashed lines, genes where Rpd3 is not bound) (B) and on the 833 genes from cluster 2 (solid lines, genes where Rpd3 is present only on promoters) and cluster 3 (C). (D) SOM clustering of Rco1, H3K36me3 and RNAPII on genes from the cluster 2 from panel “A”. Cluster 4 represents the 222 active genes with no Rco1 binding.

While it is generally accepted that Rpd3L negatively regulates specific sets of genes, the specific function of Rpd3S remains largely unaddressed. However, it is expected to be ubiquitously recruited to active genes since it interacts with methylated H3K36. In order to address the specificity of Rpd3S and Rpd3L in a systematic manner, we performed ChIP-chip experiments on myc-tagged Rpd3, Rco1 and Sds3, the last two being specific subunits of Rpd3S and Rpd3L, respectively. As shown in Figure 1A, Rpd3 binds to a large subset of promoters (Figure 1A, clusters 1 and 2). In addition, a subset of these genes also exhibit Rpd3 binding on their coding regions (Figure 1A, cluster 2). Figure 1B and 1C show the average signal of Rpd3, Rco1 and Sds3 over the genes from cluster 1 and cluster 2, respectively. The data for cluster 3, representing the genes not bound by Rpd3, are also shown. Cluster 1, which is enriched for genes previously demonstrated to be repressed by Rpd3 (genes involved in M phase (p-value 10^−8^), cell cycle (p-value 10^−9^), etc.), shows binding of both Rpd3 and Sds3 to the promoter. The presence of Rco1 is not detectable on these genes. This cluster therefore represents genes repressed by Rpd3L, which is consistent with the fact that these genes have low level of RNAPII (Figure S1A). Cluster 2, however, shows evidence for the presence of both Rpd3L and Rpd3S since all three subunits tested for are detected. Rco1 is restricted to the coding region of these genes, consistent with the fact that they are actively transcribed (Figure S1A). Sds3 is present at the promoter, which is expected since Rpd3 binds to these promoters. More surprisingly, however, some level of Sds3 is also detected on the coding region of these genes. These data - also observed with another subunit of Rpd3L (Rxt2; Figure S1B) - suggest that the large complex may play some role during transcriptional elongation, perhaps in conjunction with the small complex (see Discussion). Nevertheless, the data presented here clearly show that Rpd3S binds to the coding region of active genes.

Strikingly, these experiments also show that Rpd3S preferentially associates with genes that are also bound by Rpd3L. In fact, we found no clusters of genes where Rpd3 binds in the coding region but not in the promoter. This suggests that Rpd3S, contrary to what has been expected, does not ubiquitously bind to active genes but rather targets some of them, namely a subset of those that are bound by Rpd3L at their promoter. In order to test if Rpd3S ubiquitously binds active genes, we performed ChIP-chip experiments of RNAPII and H3K36me3, two proxies for active gene expression. Figure 1D (cluster 4) clearly shows that many genes, despite having strong enrichment for RNAPII and H3K36me3, show no evidence of Rpd3S binding. Three conclusions can be drawn from these results; firstly, they confirm that the recruitment of Rpd3S is not a general phenomenon occurring on all transcribed genes; secondly, they suggest that the methylation of H3K36 by Set2 is not providing the specificity for the recruitment of Rpd3S; and finally they suggest that the large complex may play a role in the recruitment of the small complex as Rpd3S appears to target primarily genes also bound by Rpd3L.

### Set2 is not required for Rpd3S binding to ORFs *in vivo*

The idea that H3K36me3 recruits Rpd3S through the Eaf3 subunit is well established in the literature [39]. Work done *in vitro* by several groups has clearly shown this using peptides and nucleosomal substrates [34], [35], [37], [38]. Our ChIP-chip experiments, however, clearly demonstrate that many genes harboring high levels of H3K36me3 are free of Rpd3S. We have therefore endeavored to examine whether the well characterized interaction between the Eaf3 CHD and H3K36me3 is responsible for the targeting of Rpd3S *in vivo*.

**Figure 2:**
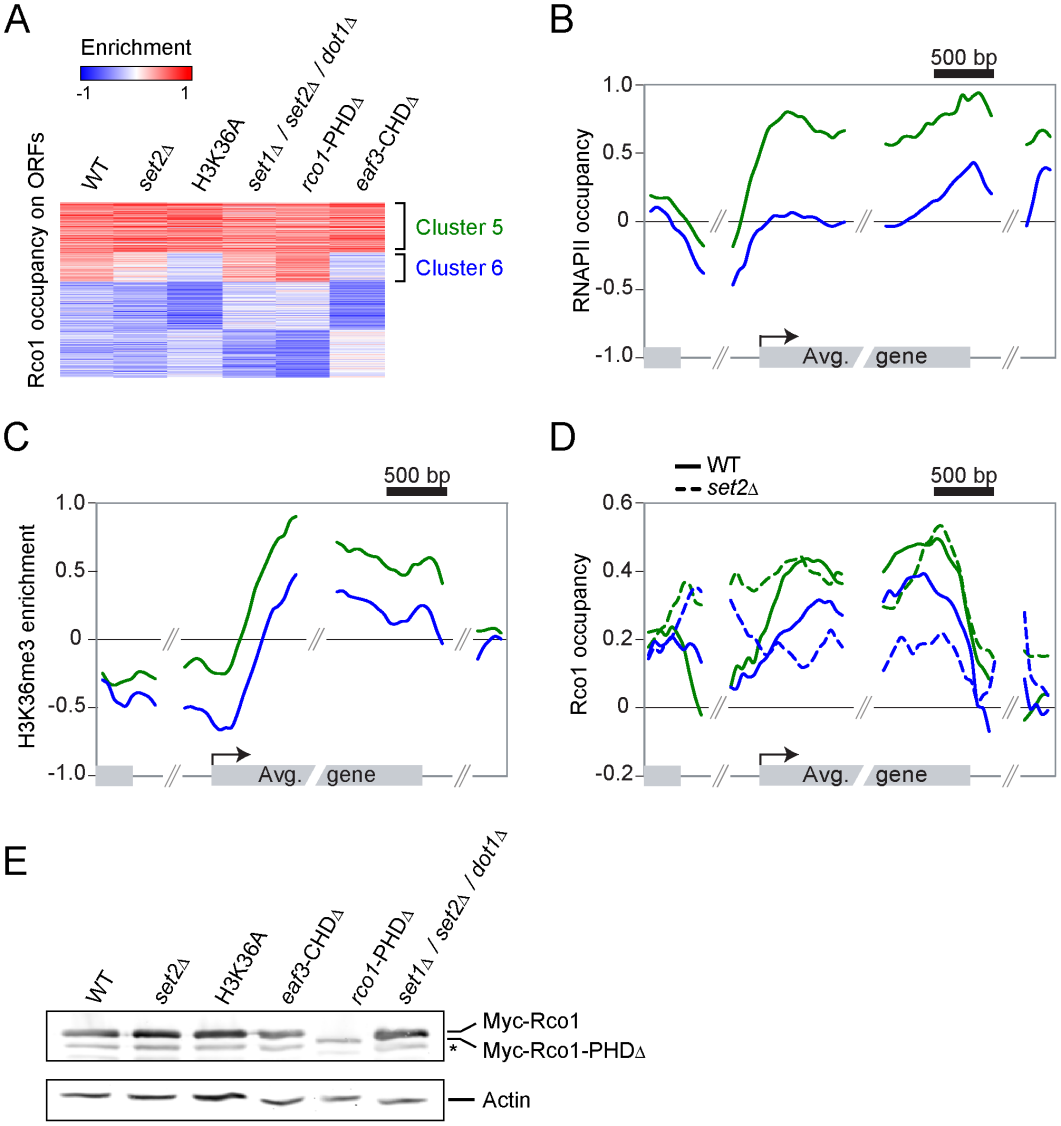
The recruitment of Rpd3S to transcribed genes does not require Set2-dependent H3K36 methylation *in vivo*. (A) SOM clustering of Rco1 enrichment on ORFs of all the 5007 genes with no missing value in WT and various mutants. (B,C) Mapping of RNAPII occupancy (B) and H3K36me3 enrichment (C) on genes contained within clusters 5 (green, 1425 genes) and 6 (blue, 841 genes) from panel “A”. (D) Mapping of Rco1 in WT (solid lines) and *set2Δ* (dashed lines) cells along genes contained within clusters 5 (green) and 6 (blue) from panel “A”. (E) Western blot showing the Rco1 protein levels in the strains used in panels A–D. Note the decreased level of the PHD-truncated Rco1 protein. The star symbol (*) indicates a degradation product.

First, we looked at Rco1 binding in a *set2Δ* mutant. Since this mutant cannot methylate H3K36, the current model predicts that Rpd3S should not bind to ORFs under these conditions. To our considerable surprise, Rco1 enrichment on ORFs in this mutant is not significantly altered for about two-thirds of Rpd3S target genes (Figure 2A, cluster 5). For other genes, Rpd3S occupancy is decreased significantly, although not completely abolished (Figure 2A, cluster 6). We next repeated these experiments in a H3K36A mutant (where lysine 36 is mutated into an alanine) with similar results (Figure 2A). The deletion of the Rco1 PHD domain had no effect on Rpd3S occupancy (despite the fact that it destabilizes the Rco1 protein; Figure 2E), while deletion of the Eaf3 CHD domain phenocopied *set2Δ* and H3K36A. Interestingly, a *set1Δ/set2Δ/dot1Δ* triple mutant (abolishing all histone methylation activity in yeast) was similar to wild type, suggesting that deletion of *SET1* and/or *DOT1* can partially suppress the *set2Δ* phenotype. Taken together, these data demonstrate that H3K36 methylation is not required for the recruitment of Rpd3S to most genes.

Since Rpd3S occupancy seems to be more dependent on H3 methylation at some genes than others, we looked more closely at clusters 5 and 6. As shown in Figure 2B and 2C, clusters 5 and 6 are markedly different with regards to transcription levels. While cluster 5 is highly transcribed (as shown by the presence of high levels of both RNAPII and H3K36me3), cluster 6 is less so. Next we looked at the distribution of Rco1 on genes contained within these clusters in all strains shown in Figure 2A. As expected from data shown in Figure 2A, Rco1 occupancy on ORFs is not (or only slightly) affected in these mutants for the cluster 5 genes, but it is reduced for the genes from cluster 6 (Figure 2D and Figure S2). In addition, for all Rpd3S-bound genes, a redistribution of Rco1 to the promoter region was observed in all mutants tested. This redistribution towards the promoter remains unexplained but correlates with our observation that histone acetylation is decreased at promoters in these same mutants (Figure S3). Collectively, these data clearly demonstrate that H3K36 methylation has no impact on Rpd3S occupancy at highly transcribed genes (genes from cluster 5). However, the methyl mark, or the ability to recognize it through the Eaf3 chromodomain, is important for optimal Rpd3S association to ORFs with lower levels of RNAPII (genes from cluster 6).

### Set2 and Rpd3S regulate the distribution of RNAPII and histone acetylation

It is known that *set2Δ* mutants, along with null mutants of Rpd3S subunits Eaf3 or Rco1, exhibit a cryptic transcription phenotype [34], [36], [37]. This phenotype is thought to be due to the improper deacetylation of transcribed ORFs by Rpd3S after each round of transcription [39] because of a lack of Rpd3S recruitment. Other groups that have characterized acetylation levels on coding regions in Set2 and Rpd3S mutants either looked at bulk chromatin by western blotting [37], or at specific genes by ChIP [34], [35], [38], [40] and have come to the conclusion that acetylation levels increase on ORFs when Set2 or Rpd3S is disrupted.

**Figure 3:**
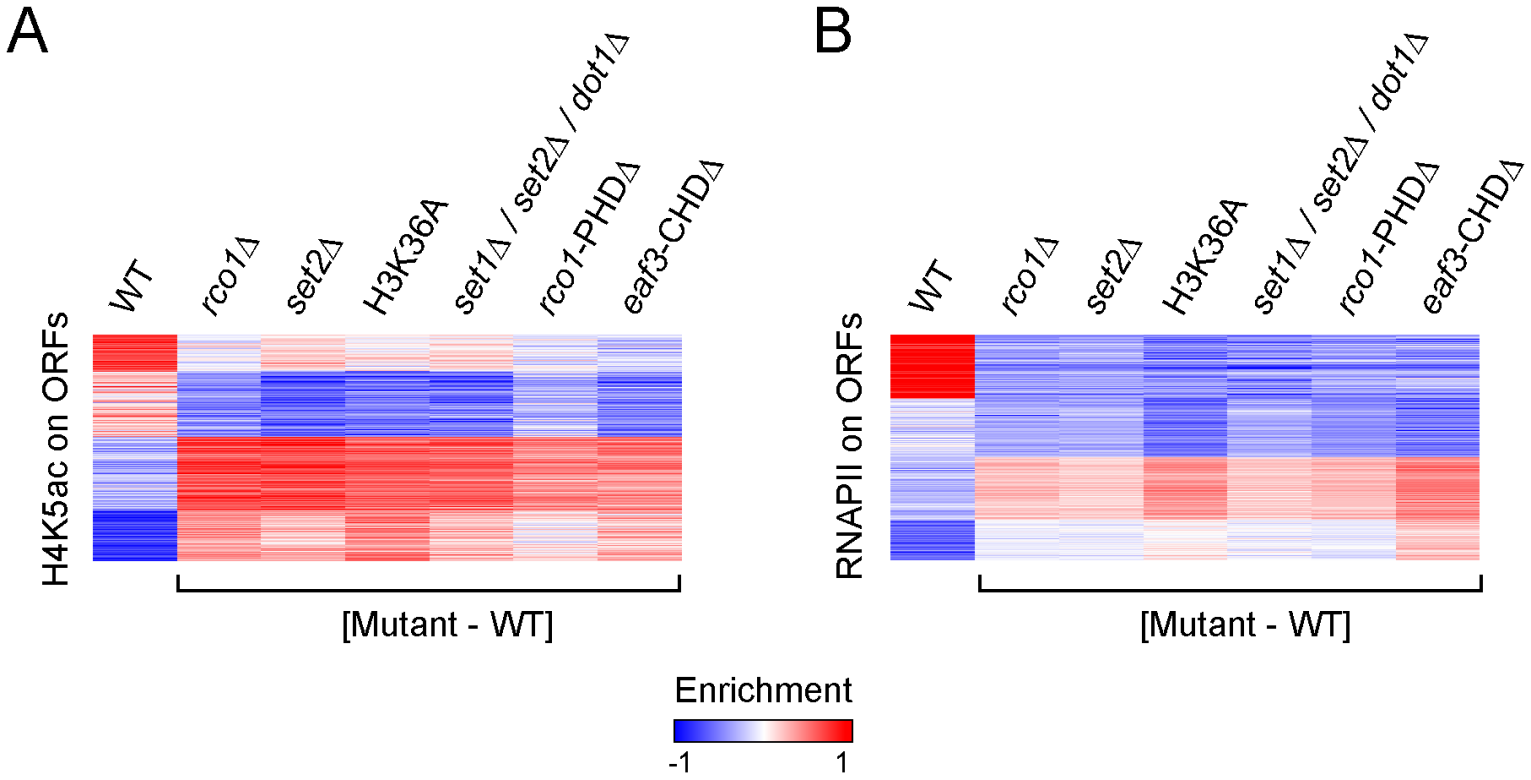
Set2-dependent H3K36 methylation is required for the function of Rpd3S. (A,B) SOM clustering of H4K5ac enrichment (A) and RNAPII occupancy (B) on ORFs of all genes in WT cells, along with the difference calculated between the enrichment observed in various mutants and WT cells.

Since we—quite surprisingly—observed, however, that Rpd3S binding to genes is mostly independent of histone methylation by Set2, we decided to test whether the activity of Rpd3S requires methylation of H3K36 by Set2. To do so, we looked at H4K5 acetylation (H4K5ac) by ChIP-chip. We used H4K5 acetylation to score for Rpd3S activity because it was shown previously to be a robust read out for Rpd3 activity in ChIP-chip assays [41]. Similar to other groups [34], [35], [38], [40], we observed decreased acetylation on promoters in *set2Δ*, H3K36A or Rpd3S mutants (Figure S3). Histone acetylation is also dramatically affected across ORFs in these mutants. As shown in Figure 3A, we observed a loss of acetylation for normally highly acetylated ORFs, and a gain in acetylation for ORFs that exhibit low levels in the wild type. Because Set2 and Rpd3S are both known to prevent cryptic initiation within ORFs, we repeated the same analyses on RNAPII ChIP-chip results, and found a similar pattern to that observed for H4K5ac, namely that ORFs with high RNAPII enrichment show decreased RNAPII levels in the absence of Set2 or Rpd3S, whereas ORFs with low RNAPII tend to display higher levels of polymerase (Figure 3B). These results clearly show that the activity of the Rpd3S complex requires methylation of H3K36 by Set2.

The effect of the loss of Rpd3S activity on histone acetylation and RNAPII distribution on ORFs is more complex than previously described. Our data indeed suggest that both histone acetylation and RNAPII occupancy are redistributed in a more even manner across the genome than expected. A plausible explanation of the genome-wide averaging of RNAPII and histone acetylation levels in Set2 and Rpd3S mutants would entail aberrant recruitment of the transcriptional apparatus to low-expression genes whose coding regions were not reset properly by a, now inactive, Rpd3S complex. Assuming a limited pool of transcription machinery in a cell, this would result in a lower abundance of RNAPII at the more active ORFs, and aberrant genomic acetylation levels.

These results, combined with the Rpd3S occupancy profiles in *rco1Δ*, *rco1-PHDΔ* and *eaf3-CHDΔ* mutants, suggest that the loss of histone H3 methylation at lysine 36 affects ORF identity through a modulation of Rpd3S deacetylase activity rather than through altered recruitment of the Rpd3S complex on coding regions as previously thought.

### Spt4 is required for the proper localization of Rpd3S

**Figure 4:**
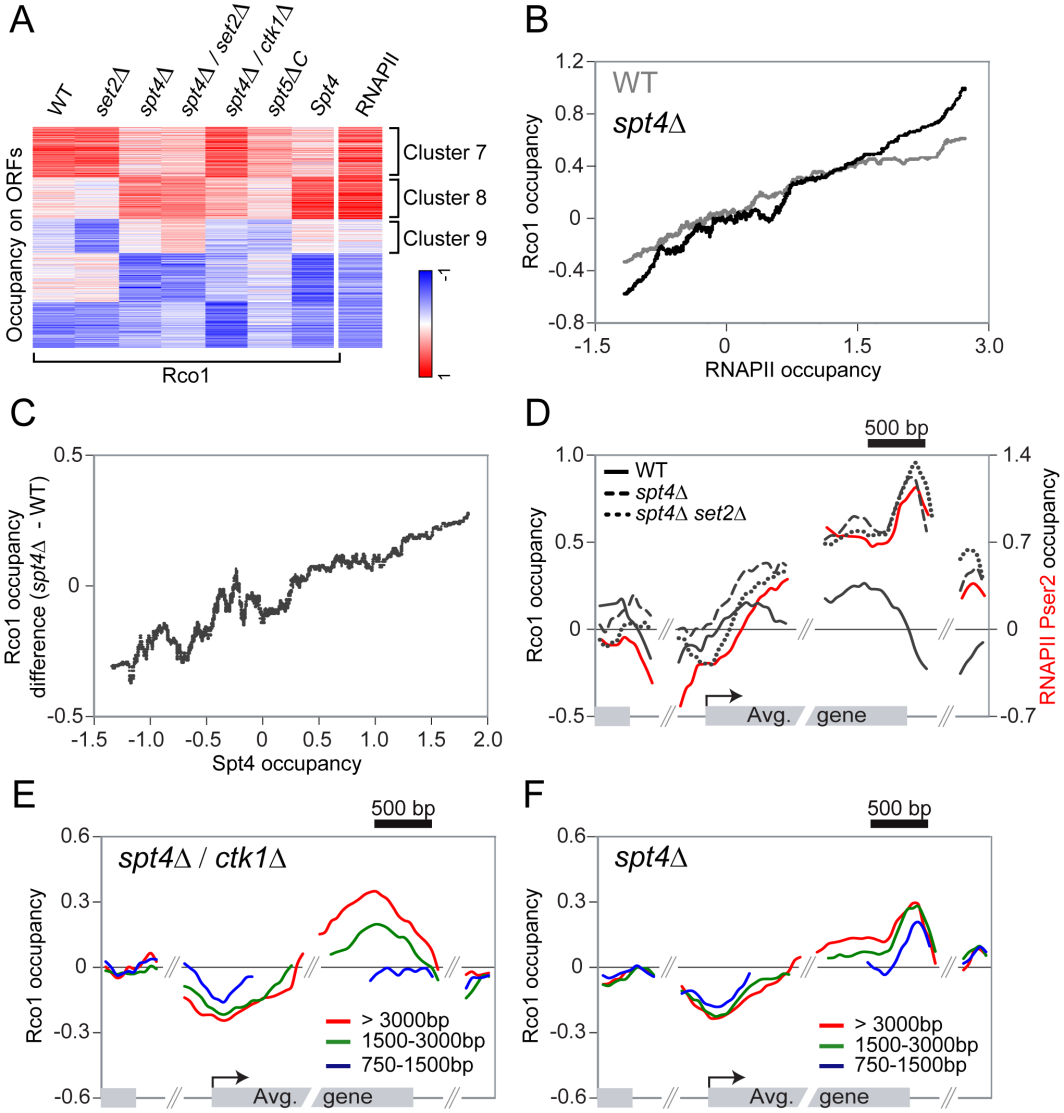
Spt4 negatively regulates the recruitment of Rpd3S. (A) SOM clustering of Rco1 occupancy on ORFs of all genes in WT, and various mutants, together with Spt4 occupancy in WT on ORFs. The RNAPII ORF occupancy in WT was added after the clustering. (B) Correlation between Rco1 occupancy and RNAPII occupancy in WT (grey) and *spt4Δ* cells (black). A sliding median window of 300 genes was applied to the data. (C) Correlation between Spt4 occupancy and the difference of Rco1 occupancy in *spt4Δ* versus WT cells. A sliding median window of 300 genes was applied to the data. (D) Mapping of Rco1 occupancy on the 953 genes contained within cluster 8 in WT (black solid line), *spt4Δ* (dashed line) and *spt4Δ*/*set2Δ* (dotted line) cells. The RNAPII phosphorylated on serine 2 enrichment is also shown (red solid line). (E,F) Mapping of Rco1 occupancy on genes grouped according to their lengths in *spt4Δ*/*ctk1Δ* (E) and *spt4Δ/set2Δ* (F) cells (red line: the 465 genes >3000bp, green line: the 1703 genes between 1500–3000bp, blue line: the 2091 genes between 750–1500bp).

Since the interaction between the Eaf3 CHD and H3K36me3 does not account for the initial recruitment of Rpd3S to active genes, we decided to look for a factor that would fulfill that role. Quan and Hartzog have shown genetic interactions between H3K36 methylation and Rpd3S with Spt5 [42]. Their data suggest that Rpd3S opposes the function of the elongation factor Spt4/5, which is the yeast ortholog of the human elongation factor DSIF [43]. DSIF negatively regulates elongation in its non-phosphorylated form, but is turned into a positive elongation factor upon phosphorylation by P-TEFb (Bur1 in yeast) [44]–[46]. The genetic interaction between Rpd3S and Spt5 led us to test whether Spt4/Spt5 is involved in the recruitment of Rpd3S. We therefore performed ChIP-chip experiments of Rco1 in *spt4Δ* cells (the deletion of *SPT5* is lethal). As shown in Figure 4A, deletion of *SPT4* leads to massive changes in Rco1 binding across the genome. Notably, the effect is far more dramatic compared to the deletion of *SET2* (Figure 4A). Importantly, the level of RNAPII observed on these genes is not significantly affected in the mutant, ruling out the possibility that the effect is solely due to a reshuffling of the transcriptome (Pearson correlation = 0.94, Figure S4A). The deletion of *SPT4* causes a decrease of Rco1 binding at some transcribed genes normally strongly associated with Rco1 (Figure 4A, cluster 7), as well as an increase at others where Rco1 is otherwise only found at low levels (cluster 8). Deletion of *SPT4* even causes a slight increase of Rco1 occupancy at genes where it is normally undetectable (cluster 9). Overall, these effects lead to a distribution of Rpd3S that correlates better with RNAPII occupancy than in wild type cells (Figure 4B). To test the possibility that, in the absence of Spt4, Rpd3S is recruited via H3K36 methylation, we profiled Rco1 binding in a *spt4Δ/set2Δ* double mutant. As shown in Figure 4A, deleting both *SPT4* and *SET2* leads to a similar Rpd3S localization phenotype compared to the single *spt4Δ* mutant, giving further evidence that Set2 does not play a large role in Rpd3S recruitment, even in the absence of Spt4.

### Spt4 negatively regulates the association of Rco1 with transcribed genes

To distinguish the direct effect of the loss of *SPT4* from eventual indirect effects of the mutation, we localized Spt4 in wild type cells by ChIP-chip using a strain carrying a myc-tagged *SPT4* gene. Spt4 associates with genes in a manner that correlates with levels of RNAPII (Pearson correlation = 0.84) suggesting that DSIF acts as a general elongation factor. Moreover, it is present across the whole ORF, indicating that it travels with RNAPII, but is further enriched in the 3′ end of genes (Figure S4B), suggesting that it may also regulate the elongation-termination transition, as shown by others [47]–[49]. This is also consistent with the fact that Spt5 interacts with components of the capping and termination machineries [50]. Even more interestingly, the more a gene is occupied by Spt4 in wild type cells, the more Rco1 we detect in the *spt4Δ* mutant (Figure 4C), suggesting that the direct effect of the loss of Spt4 is an increase in Rpd3S binding (as observed for cluster 8). Consequently, the decrease in occupancy observed in cluster 7 is most likely indirect since Spt4 is barely detectable at these genes (Figure 4A). Similarly, the level of Rco1 increases dramatically in the *spt4Δ* mutant on cluster 4 genes (from Figure 1D), representing transcribed genes highly occupied by Spt4 where Rco1 is absent in wild type cells (Figure S4C). In general, genes with a higher Spt4/RNAPII ratio tend to have less Rco1 than genes with lower Spt4/RNAPII ratios (Pearson correlation = −0.42, Figure S4D). Taken together, these data suggest that Spt4 acts as a negative regulator of Rpd3S binding and that its presence prevents the HDAC from freely associating with transcribed genes. This model is in agreement with genetic data showing that Rco1 opposes the function of Spt4/5 [42].

We then looked at the distribution of Rpd3S on transcribed genes where Spt4 is also bound (cluster 8) and found strong differences between the wild type and *spt4Δ* mutants. While Rco1 occupies the whole ORF at a constant level in wild type cells, it accumulates to abnormally high levels towards the 3′ end of the gene in *spt4Δ* cells (Figure 4D, dashed line). This binding pattern is also found in the *spt4Δ/set2Δ* double mutant (compare dashed and dotted lines in Figure 4D). The occupancy profile of Rco1 in a *spt4Δ* mutant shows a strong similarity to the occupancy profile of RNAPII with a CTD phosphorylated at Ser2 (Figure 4D, red solid line). This led us to hypothesize that CTD phosphorylation by Ctk1 (the major serine 2 kinase) might be implicated in Rpd3S recruitment in the absence of Spt4/5. To test this hypothesis, we profiled Rco1 occupancy in a *spt4Δ/ctk1Δ* background. Surprisingly, *ctk1Δ* partially suppressed the *spt4Δ* Rpd3S binding pattern phenotype (Figure 4A) leading to an intermediate binding profile between wild type and *spt4Δ*. Looking at it more closely, we observed that short genes fail to accumulate Rco1 in *spt4Δ/ctk1Δ* cells (Figure 4E), whereas Rco1 is observed at genes irrespective of their length in wild type (Figure S5) or *spt4Δ* cells (Figure 4F), suggesting that Rpd3S accumulates more slowly in *spt4Δ*/*ctk1Δ* cells. We therefore conclude that in the absence of Spt4, Rpd3S binds to the RNAPII CTD and that the phosphorylation of the CTD at serine 2 contributes to that phenomenon.

### Phosphorylation of the RNAPII CTD triggers the association of Rpd3S to active genes

**Figure 5:**
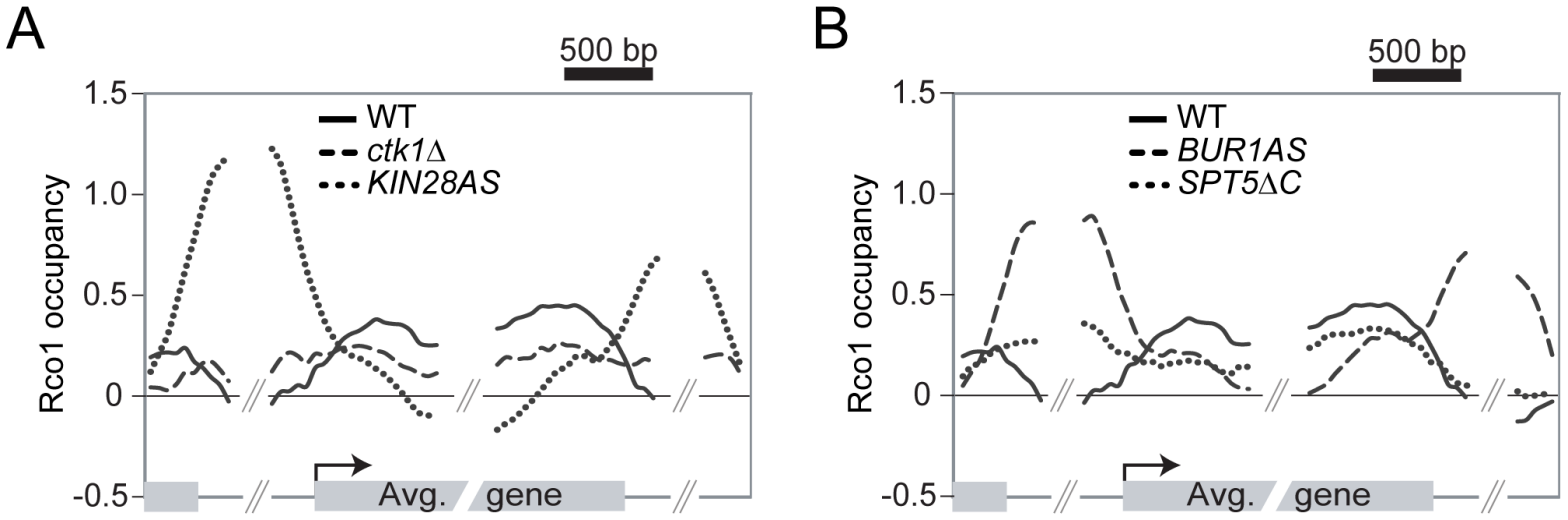
Phosphorylation of the RNAPII and Spt5 CTD are important for the Rpd3S recruitment on genes. (A) Mapping of Rco1 occupancy on the 2089 genes contained within clusters 7 and 8 in WT (solid line), *ctk1Δ* (dashed line), and *kin28AS* (dotted line) cells. (B) As in ‘A’ using the *bur1AS* (dashed line) and *spt5ΔC* (dotted line) cells.

The data presented above demonstrate that Spt4 negatively regulates the association of Rpd3S to highly transcribed genes. The data also suggest that phosphorylation of the RNAPII CTD, notably at serine 2, plays some role in the recruitment of Rpd3S to active genes in the absence of Spt4. We next tested whether the phosphorylation of the CTD is implicated in the association of Rpd3S with transcribed genes in wild type cells and performed ChIP-chip experiments of Rco1 in mutants for the known CTD kinases. As shown in Figure 5A, deletion of *CTK1*, the major serine 2 kinase, has a clear effect on Rco1 occupancy (compare solid line with dashed line). To test the effect of serine 5 and 7 phosphorylation, we used a strain carrying ATP-analog-sensitive alleles of *KIN28* since the deletion of the *KIN28* gene is lethal. As shown in Figure 5A (dotted line), inhibition of Kin28 has a dramatic effect on Rco1 occupancy. Indeed, no Rco1 can be detected on ORFs in that mutant. This clearly demonstrates that phosphorylation of serine 5 and/or 7 by Kin28 is a major element in the recruitment of Rpd3S to active genes. These results are supported by data from the Hinnebusch lab who have shown that Rpd3S interacts with the phosphorylated form of RNAPII and has high affinity for doubly phosphorylated (serine 2/5) CTD peptides *in vitro* [51]. Interestingly, CTD peptides carrying a single phosphate group on serine 5 or serine 2 respectively have a much weaker or no affinity to Rpd3S compared to doubly phosphorylated CTD peptides. Also noteworthy is the fact that Rpd3S is redistributed to promoter regions when Kin28 is inactive (Figure 5A). As we will be discuss below, this might be due to a decrease in H3K36 methylation in that mutant, most likely due to a defective Bur1/2 recruitment, as suggested by [52] and [53], leading to a defect in Rad6 phosphorylation. Our genome-wide data, together with these *in vitro* experiments, suggest that phosphorylation of the RNAPII CTD stimulates the recruitment of Rpd3S to transcribed genes while the elongation factor DSIF counteracts this recruitment.

### Phosphorylation of Spt5 by Bur1 negatively regulates the activity of DSIF on Rpd3S recruitment

In mammalian cells, DSIF is phosphorylated by Cdk9, a cyclin-dependent kinase associated with the elongation factor P-TEFb [54]. Cdk9 also phosphorylates the RNAPII CTD on serine 2 as well as other proteins including NELF. In yeast, the function of Cdk9 is fulfilled by two distinct kinases. Ctk1 mainly phosphorylates the RNAPII CTD and Bur1 targets Spt5 and Rad6 [55]–[57]. Inactivation of Bur1 has modest effects on phosphorylation of the RNAPII CTD, as shown by western blot and by ChIP-chip experiments (data not shown; see also [52], [57], [58]). Because Bur1 phosphorylates Spt5, the partner of Spt4, we tested the effect of inactivating Bur1 activity on Rco1 recruitment. Not surprisingly, inhibiting Bur1 using an ATP-analog-sensitive strategy (*bur1*AS) has a profound effect on Rco1 occupancy. In the absence of a functional Bur1, Rco1 is depleted from the coding region and redistributed to promoter regions (Figure 5B, dashed line) as was observed in the Kin28 mutant while the RNAPII level is mostly unchanged (data not shown). Deleting the CTD of Spt5 (*spt5ΔC*), the region phosphorylated by Bur1, caused a similar, although milder, phenotype (Figure 5B, dotted line). The stronger effect observed in the Bur1 inactivation experiment, compared to truncation of Spt5, is most likely due to the fact that Bur1 has additional targets. For example, Bur1 phosphorylates Rad6, an event that is required for the methylation of H3K4 and K79 by Set1 and Dot1 respectively [59]–[61]. In a triple mutant for Set1, Set2 and Dot1, we observed a shift of Rpd3S toward intergenic regions (Figure S2B), suggesting that the redistribution of Rpd3S to promoter regions in Bur1-impaired cells is at least in part a consequence of Bur1's activity on Rad6. Nevertheless, on the coding region, where our *spt4Δ* data showed that DSIF negatively regulates Rpd3S binding, we observed a clear decrease in Rco1 occupancy in both *bur1*AS and *spt5ΔC*. These data strongly suggest that phosphorylation of Spt5 by Bur1 negatively regulates the activity of DSIF on Rpd3S recruitment. While we cannot completely rule out the possibility that some of the effect observed in the *bur1*AS strain is due to an effect of Bur1 on the RNAPII CTD, our data on *spt5ΔC* rather suggest that Bur1 regulates Rpd3S recruitment by regulating DSIF. Interestingly, phosphorylation of Spt5 by Bur1 was previously shown to stimulate its activity as an elongation factor while our data suggest that it inhibits its activity as a negative regulator of Rpd3S recruitment. It will therefore be interesting to see whether these activities are linked.

## Discussion

**Figure 6:**
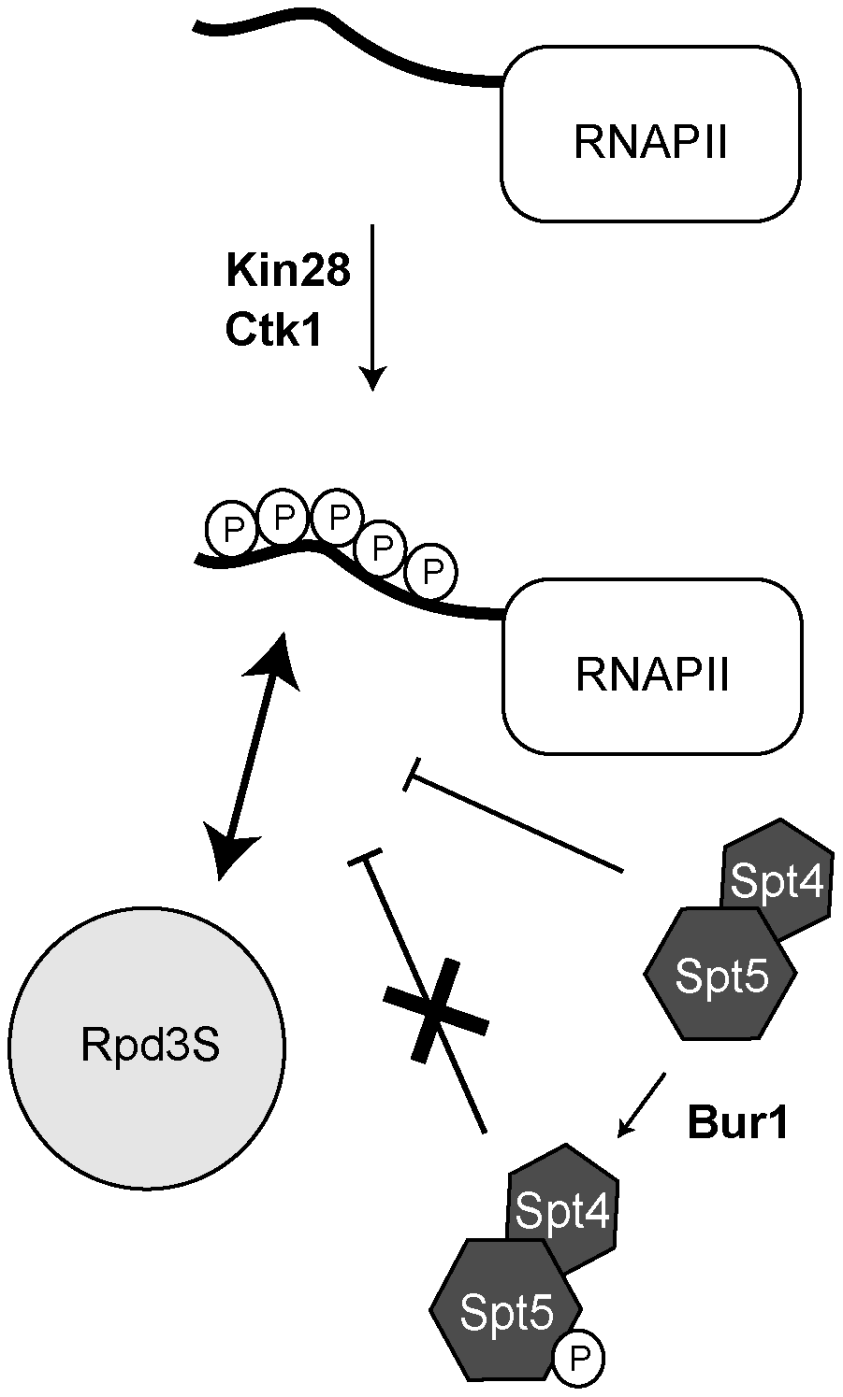
A model summarizing how Rpd3S is recruited to active genes. Phosphorylation of the RNAPII CTD by Kin28 and Ctk1 stimulates the recruitment of Rpd3S to the CTD. DSIF (Spt4/Spt5) counteracts the recruitment of Rpd3S but its phosphorylation by Bur1 alleviates its repressing activity.

The Rpd3S complex is recruited to the coding region of transcribed genes where it represses cryptic transcription by deacetylating histones in the wake of the elongating polymerase, therefore resetting chromatin to its pre-transcriptional state. The recruitment of Rpd3S to transcribed regions was thought to be mediated by the interaction between the Eaf3 CHD and H3K36me. However, the interaction of Rpd3S on the coding regions of actively transcribed genes has never been directly demonstrated *in vivo*. Here, we build on that model and show that: 1) Rpd3S targets a subset of transcribed genes; 2) Rpd3L is present at the promoter of genes where Rpd3S is bound; 3) The activity of Rpd3S requires methylation of H3K36 by Set2 but its association with active genes does not; 4) The recruitment of Rpd3S on ORFs requires the phosphorylation of the RNAPII CTD by Kin28 and Ctk1; and 5) The DSIF elongation complex counteracts the recruitment of Rpd3S to transcribed genes, a phenomenon that is regulated by the phosphorylation of Spt5 by Bur1. We therefore propose a model where the opposing effects of CTD phosphorylation and DSIF on Rpd3S recruitment together allow for the complex occupancy profile of that HDAC *in vivo* (Figure 6).

### The distribution of Rpd3S along the genome is more complex than expected

Because H3K36 methylation correlates with transcription, it is generally accepted to be present at all transcribed genes; Rpd3S was therefore expected to follow the same pattern. Our finding that many transcribed genes do not show any signs of Rpd3S binding despite the presence of H3K36me3 was therefore a considerable surprise. This has important implications since it suggests that not all genes are equally protected against cryptic initiation. Other mechanisms exist that prevent cryptic transcription [62], so it will be interesting to investigate how these various activities share the labor of protecting the genome from aberrant transcription.

### Does Rpd3L play a role in the targeting of Rpd3S?

Another peculiarity of Rpd3S is that it is preferentially enriched on the coding regions of genes where Rpd3L is found on the promoter: we rarely find Rpd3S on genes where Rpd3L is not present. The presence of Rpd3L on the promoter of active genes is counterintuitive given its role as a co-repressor but it has nevertheless being observed before [17]. The co-occurrence of Rpd3L at promoter and Rpd3S on the ORFs of the same genes suggests that Rpd3L may play a role in targeting Rpd3S. One possibility would be that Rpd3S emerges from Rpd3L during the transition from initiation to elongation. The transition to elongation may trigger the exchange of subunits to transform Rpd3L into Rpd3S. Our finding that DSIF and RNAPII CTD phosphorylation are involved in the proper recruitment of Rpd3S to chromatin suggests that they may play a role in that process.

Unexpectedly, we also observe Rpd3L on the coding regions of the genes where Rpd3S is also present. This last result was surprising in that it does not agree with the generally accepted function of Rpd3L as a promoter-recruited co-repressor. These data argue for a new model where subunits of both Rpd3L and Rpd3S coexist on actively transcribed coding regions. According to the model described above, the presence of Rpd3L subunits may reflect an imperfect exchange of subunits during the transition from initiation to elongation. Alternatively, it may reflect the presence of an Rpd3 “super large” complex that contains subunits of both Rpd3S and Rpd3L. In such a model, Rpd3S (as we normally see it) would only exist in solution and would represent a module that joins Rpd3L after initiation. This hypothesis is supported by data provided by Collins et al. [63] who combined and re-analyzed previously published mass spectrometry data [64], [65] to generate a high-accuracy yeast protein interaction dataset. They found that several subunits of Rpd3L can be co-purified using Rco1 as bait. Reciprocally, subunits of Rpd3S can be co-purified with Rpd3L subunits used as baits. These data argue that some interactions between Rpd3S and Rpd3L do exist in the cell. Since the complexes analyzed in these studies were purified from the soluble cellular fraction, the relative low abundance of these inter-complex interactions may be explained by the fact that these complexes normally exist together only on chromatin. The recent development of techniques to purify protein complexes from chromatin [66], [67] may help testing these intriguing possibilities.

### The role of the Eaf3 CHD in Rpd3S function

We show that the recruitment of Rpd3S to active genes does not require H3K36 methylation, Set2 or the Eaf3 chromodomain. Interestingly, Rco1 binding is not abolished in a triple deletion mutant for all the known yeast HMTs (Set1, Set2 and Dot1), ruling out the possibility that the previously described interaction between H3K4me3 and Eaf3 [38] (or an eventual interaction with methylated H3K79) is compensating for the loss of H3K36 methylation in *set2Δ* and H3K36A mutants. Moreover, although the methylation state of H3K36 does not affect Rpd3S binding at highly transcribed genes, it has an effect on the occupancy of Rpd3S at ORFs showing lower transcription levels. This suggest that, despite not providing the main recruitment signal, H3K36 methylation provides some stabilizing effect on the association of Rpd3S with chromatin. While Rpd3S recruitment is largely independent on H3K36 methylation, the activity of Rpd3S, however, depends on the integrity of the Set2/Rpd3S pathway, namely Set2-dependant H3K36 methylation, the Rco1 PHD and the Eaf3 CHD. We therefore propose that the main function of H3K36 methylation is to “activate” Rpd3S after it has been recruited by virtue of its association with the RNAPII CTD. This may involve the anchoring of Rpd3S on chromatin via the Eaf3-H3K36me interaction.

### RNAPII phosphorylation by Kin28 and Ctk1 is required for the recruitment of Rpd3S

Our data clearly show that phosphorylation by Kin28 is absolutely required for the association of Rpd3S with the coding region of transcribed genes. This suggests that phosphorylation of serine 5 provides the signal for the association of Rpd3S with early elongating RNA polymerases II molecules. Since Kin28 was recently shown to phosphorylate serine 7 in addition to serine 5, we cannot rule out the possibility that serine 7 phosphorylation also contributes to the binding of Rpd3S to RNAPII. Using a *ctk1Δ* strain, we also show that phosphorylation of serine 2 also contributes -although to a lesser extend than serine 5- to the association of Rpd3S with transcribed genes. These results are in perfect agreement with a recent paper from the Hinnebusch group who showed that RNAPII CTD peptides harboring a phosphate group on both serines 5 and 2 have a high affinity for Rpd3S *in vitro* while a single phosphate on serine 5 has a reduced affinity [51]. More importantly, they were not able to detect any interaction of Rpd3S with non-phosphorylated CTD peptides, and peptides carrying a single phosphate on serine 2 barely show any affinity with Rpd3S [51]. We therefore propose that Rpd3S is recruited to active genes via interaction with the phosphorylated RNAPII CTD. However, this complex remains inactive until it has been “anchored” on chromatin via H3K36 methylation. As discussed above, this phenomenon does not appear to occur equally on all genes. Negative regulation of these interactions by the DSIF elongation complex modulates association of Rpd3S with genes, therefore creating situations where active genes with high level of Spt4 carry much less Rpd3S than expected from their transcriptional level.

### DSIF is a negative regulator of Rpd3S recruitment

Interestingly, we found that deletion of *SPT4*, a subunit of DSIF, has a profound effect on Rpd3S occupancy *in vivo*. In the absence of Spt4, Rpd3S associates with the genome in a manner that correlates better with transcription than in wild type cells. This suggests that DSIF is involved in regulating the amount of Rpd3S on transcribed genes. DSIF appears to prevent the association of Rpd3S to a subset of transcribed genes, and even at genes occupied by Rpd3S, DSIF also plays a role since it prevents the hyper-accumulation of the HDAC in the 3′end of the gene. How exactly an elongation factor can regulate the association of a HDAC with elongating RNAPII remains obscure but we envision several mechanisms by which it may operate: 1) Rpd3S may directly or indirectly interact with DSIF; 2) The association of DSIF with the elongation complex may prevent the association of the HDAC; 3) DSIF may modulate the speed of the elongation complex in a way that makes it less favorable for Rpd3S binding; 4) DSIF may also impinge on the phosphorylation of the RNAPII CTD. More complex mechanisms may also be envisioned. For instance, Pin1, a proline isomerase that may modify the RNAPII CTD, binds to both, phosphorylated DSIF [68] and Rpd3/Sin3 [69]. CTD isomerisation may therefore be involved in the regulation of the recruitment of Rpd3S.

### Bur1 downregulates the activity of DSIF

In human and yeast, DSIF is regulated by phosphorylation of the CTD of its Spt5 subunit. In yeast, this phosphorylation is mediated by the Bur1 cyclin-dependent kinase [56], [57]. We therefore tested whether phosphorylation of Spt5 by Bur1 affects Rpd3S occupancy *in vivo*. As expected, both the catalytic inactivation of Bur1 and the removal of its substrate (by deleting the CTD of Spt5) have a dramatic impact on Rpd3S occupancy. Both these mutants indeed show a decreased in Rpd3S occupancy along genes. These data suggest that phosphorylation of Spt5 by Bur1 negatively regulate the activity of DSIF on Rpd3S recruitment. In addition, the inactivation of Bur1 also causes Rpd3S to redistribute to promoter regions, a phenomenon that we also observed in *set2Δ* cells. Since Bur1 phosphorylates Rad6, which is also required for H3K36 methylation by Set2, it appears likely that this redistribution in Bur1 mutants is due to its effect on H3K36 methylation via Rad6. Why a lack in H3K36 methylation leads to an association of Rpd3S with promoters remains unknown, but is in agreement with the previous observation that histone acetylation decreases at promoters in these mutants.

Taken together, our in-depth analysis of Rpd3S genomic occupancy has revealed several key insights about its recruitment to genes *in vivo*. Our study highlights a complex network of protein-protein interactions mediated by phosphorylation of several substrates by at least three kinases. Interestingly, there is previous evidence in the literature linking these kinases to the function of DSIF. First, P-TEFb (Cdk9) and Bur1 can phosphorylate DSIF [56], [57], [70]. Second, Kin28, Ctk1 and Bur1 exhibit synthetic genetic interactions with Spt4 and Spt5 [71]. And third, the recruitment of Bur1 is stimulated by Kin28 [52], [72]. Finally, both DSIF and Kin28 have been shown to stimulate the recruitment of the Paf1 complex to the elongation complex [57], [73]. A lot more work will be required before we completely understand the interplay between these factors and Rpd3S.

## Materials and Methods

### Strains, cell growth, and crosslinking conditions

All strains used in this study are listed in Table S1. All strains were grown in 50mL of YPD to an OD_600_ of 0.6–0.8 before crosslinking, unless otherwise indicated. For ChIP-chip, most strains were crosslinked with 1% formaldehyde for 30 min at room temperature on a wheel. The Rco1-9myc strains were crosslinked with 1% paraformaldehyde for 30 min at room temperature followed by 90 min at 4°C on a wheel. ATP analog-sensitive strains were treated with 6 µM of NAPP1 for 15 minutes prior to crosslinking.

### Chromatin immunoprecipitation and antibodies

ChIP experiments were performed as per [74], with minor modifications. For myc-tag ChIP, we used 5µg of 9E11 antibody coupled to 2×10^7^ pan-mouse IgG DynaBeads (Invitrogen) per sample. For histone H4K5 acetylation ChIP, we used 4µL of a rabbit serum (Upstate 07-327) coupled to 2×10^7^ protein G DynaBeads per sample. Histone H3K36me3 was immunoprecipitated with 4µL of antibody (Abcam Ab9050) coupled to 2×10^7^ protein G DynaBeads per sample. Histone H4 levels were assayed with 2µL of an antibody raised against recombinant yeast histone H4 (a gift from Alain Verreault) coupled to 2×10^7^ protein G DynaBeads per sample. RNAPII ChIPs were done using 2µL of 8WG16 antibody coupled to 2×10^7^ pan-mouse IgG DynaBeads per sample. Note that in our ChIP-chip assays, 8WG16 generates profiles that are nearly identical as using a tagged RNAPII (data not shown). 8WG16 is therefore used here to measure total RNAPII levels on genes. RNAPII CTD serine 2 phosphorylation was assayed using 5µL of H5 antibody (Covance Research MMS-129R-200) coupled to 2×10^7^ protein G DynaBeads per sample.

The microarrays used for location analysis were purchased from Agilent Technologies (Palo Alto, California, United States) and contain a total of 44,290 Tm-adjusted 60-mer probes covering the entire genome for an average density of one probe every 275 bp (±100 bp) within the probed regions (catalog # G4486A and G4493A). Myc-tag ChIPs were hybridized against ChIPs from isogenic strains that did not contain the tag as controls. Acetylation, RNAPII and histone H4 ChIPs were hybridized against a sample derived from 400ng of input (non-immunoprecipitated) DNA. Acetylation levels were normalized to histone H4 levels by subtracting Log_2_ (Histone H4/input) from Log_2_ (H4K5 acetyl/input). All microarray experiments described in this work are listed in Table S2, the processed data are available in Datasets S1, S2, S3, S4, and the raw data have been deposited into the GEO database (Accession # GSE22636).

### Data analysis

The data were normalized and biological replicates were combined using a weighted average method as described previously [74]. The log_2_ ratio of each spot of combined datasets was then converted to Z-score, similar to Hogan et al. [75], to circumvent the large differences in the immunoprecipitation efficiencies of the different factors. Visual inspection of the Z-scores was carried out on the UCSC Genome Browser (http://genome.ucsc.edu/). All data analyses described here were done using data from protein-coding genes longer than or equal to 500 bp. Median Z-score values for promoter and complete length of each annotation from SGD (version Feb. 02 2008) were calculated without interpolation (Dataset S5) and used in our clustering and Pearson correlation analyses. Promoters are defined as the shortest of either 250bp or half the intergenic region (half-IG) relative to the reference gene's 5′ boundary. Self-organizing map (SOM) clustering was done with the Cluster software [76] and visualized with Java Treeview [77]. Only genes with no missing value were used for clustering.

Gene mapping was performed as in Rufiange et al. [78] on selected groups of genes described in the text. Briefly, data were mapped onto the 5′ and 3′ boundaries in 50 bp windows for each half-gene and adjacent half-IG regions. A sliding window of 300 bp was then applied to the Z-scores to smooth the curve.

## Supporting Information

Dataset S1 A UCSC Genome Browser-ready file containing all the log2 enrichment ratios for all the Rco1 localization data from this study.
(5.89 MB CDX)

Dataset S2 A UCSC Genome Browser-ready file containing all the log2 enrichment ratios for all the H3K5ac localization data from this study.
(4.06 MB GZ)

Dataset S3 A UCSC Genome Browser-ready file containing all the log2 enrichment ratios for all the RNAPII localization data from this study.
(4.52 MB GZ)

Dataset S4 A UCSC Genome Browser-ready file containing all the log2 enrichment ratios for all the experiments not already in Datasets S1, S2, S3, S4.
(2.26 MB GZ)

Dataset S5 A file containing the average Z-score calculated on each genome annotation.
(4.88 MB ZIP)

Figure S1 A complement to Figure 1. Average signal of RNAPII, Rxt2 and Sds3 over the genes of the 3 clusters. (A) Mapping of RNAPII, Rxt2 and Sds3 occupancy on genes contained within clusters 1 (solid line, 954 genes), 2 (dashed line, 833 genes) and 3 (dotted line, 1906 genes).
(0.23 MB PDF)

Figure S2 A complement to Figure 2. The recruitment of Rpd3S to transcribed genes does not require Set2-dependent H3K36 methylation *in vivo*. (A–D) Mapping of Rco1 occupancy on genes contained within clusters 5 (green, 1425 genes) and 6 (blue, 841 genes) from Figure 2 in WT and various mutants.
(0.45 MB PDF)

Figure S3 A complement to Figure 3. Deletion of Set2 or disruption of Rpd3S causes a general decrease in histone acetylation at promoters. SOM clustering of the enrichment of H4K5ac on promoters of all genes in WT cells along with the difference calculated between the enrichment observed in WT and various mutants.
(0.17 MB PDF)

Figure S4 A complement to Figure 4. Spt4 negatively regulates the recruitment of Rpd3S. (A) Mapping of RNAPII occupancy in WT (solid lines) and *spt4Δ* (dashed lines) cells on genes contained within clusters 7 (red, 1136 genes), 8 (blue, 953 genes ), 9 (green, 771 genes ), and the non-identified following clusters 10 (purple, 1031 genes) and 11 (gold, 1109 genes) of the Figure 4. (B) Mapping of Spt4 occupancy on genes binned by their RNAPII occupancy. (C) Mapping of RNAPII (gold), Spt4 (blue) and Rco1 (red) occupancy in WT (solid line) and *spt4Δ* (dashed line) cells on the 222 genes contained within clusters 4 from Figure 1D. (D) Anti-correlation between Rco1 occupancy and the difference between Spt4 and RNAPII occupancy in WT cells measured on the 2286 transcribed genes (RNAPII>0). A sliding median window of 300 genes was applied to the data.
(0.55 MB PDF)

Figure S5 Rpd3S ORF occupancy level is gene length-dependant in a *spt4Δ*/*ctk1Δ* mutant. (A–D) Mapping of Rco1 occupancy on genes grouped according to their lengths in WT (A), *set2Δ* (B), *ctk1Δ* (C), *spt4Δ* (D), *spt4Δ/set2Δ* (E), and *spt4Δ*/*ctk1Δ* (F) cells (red line: the 465 genes >3000bp, green line: the 1703 genes between 1500–3000bp, blue line: the 2091 genes between 750–1500bp).
(0.39 MB PDF)

Table S1 A list of all the yeast strains used in this study.
(0.09 MB DOC)

Table S2 A list of all the microarray hybridizations done in this study.
(0.07 MB DOC)
