# Supplementary figures and images for: DSIF and RNA Polymerase II CTD Phosphorylation Coordinate the Recruitment of Rpd3S to Actively Transcribed Genes
Source: PLoS Genet. 2010 Oct 28;6(10):e1001173. doi: 10.1371/journal.pgen.1001173 (PMC2965751; doi:10.1371/journal.pgen.1001173)

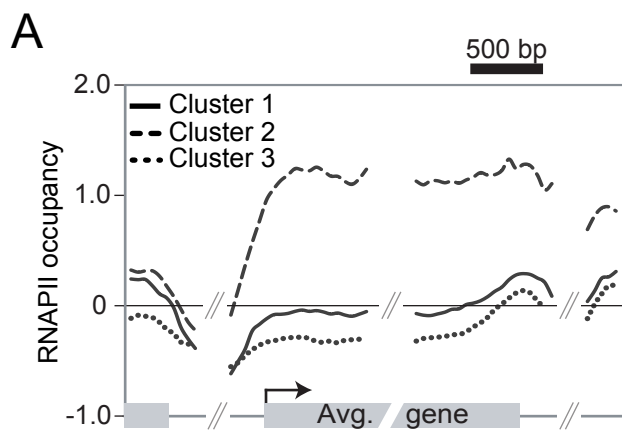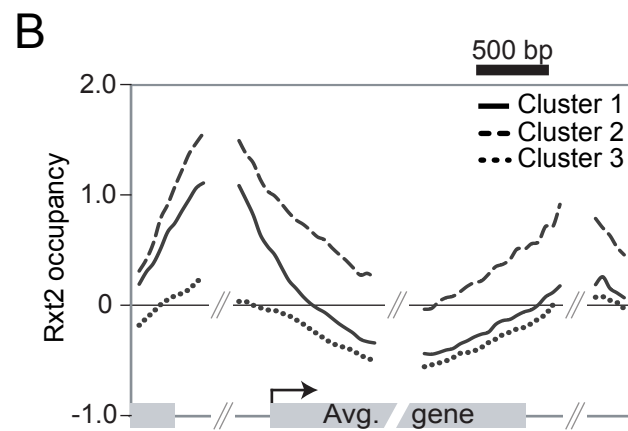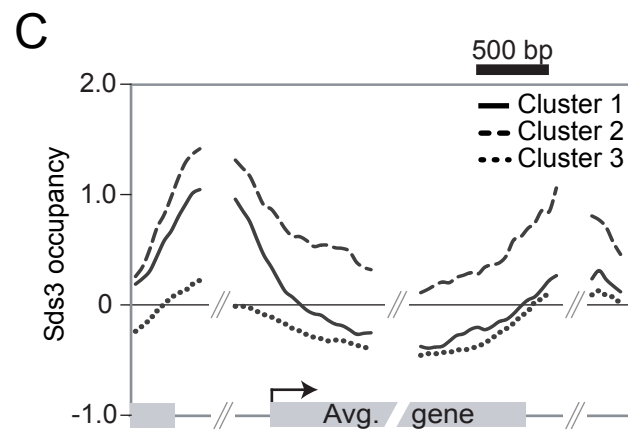

FigS1

Supplement: Figure S1 — A complement to Figure 1. Average signal of RNAPII, Rxt2 and Sds3 over the genes of the 3 clusters. (A) Mapping of RNAPII, Rxt2 and Sds3 occupancy on genes contained within clusters 1 (solid line, 954 genes), 2 (dashed line, 833 genes) and 3 (dotted line, 1906 genes). (0.23 MB PDF) [file pgen.1001173.s006.pdf]

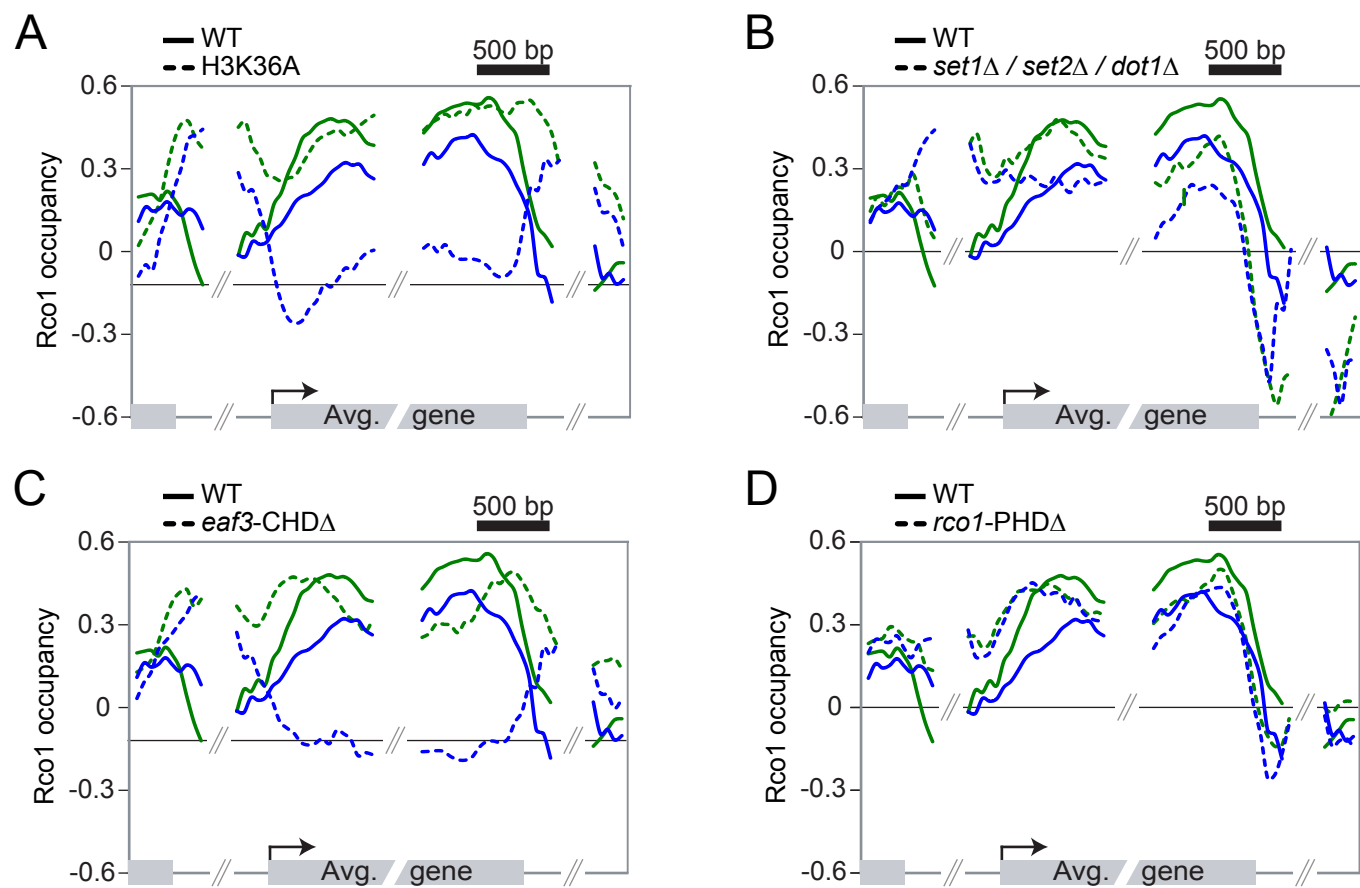

FigS2

Supplement: Figure S2 — A complement to Figure 2. The recruitment of Rpd3S to transcribed genes does not require Set2-dependent H3K36 methylation in vivo. (A–D) Mapping of Rco1 occupancy on genes contained within clusters 5 (green, 1425 genes) and 6 (blue, 841 genes) from Figure 2 in WT and various mutants. (0.45 MB PDF) [file pgen.1001173.s007.pdf]

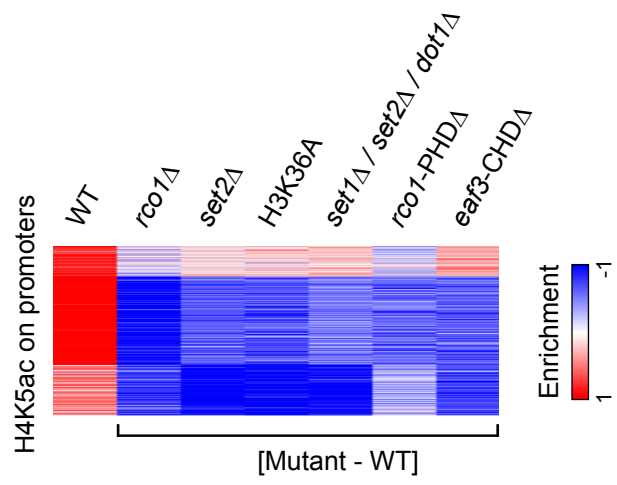

FigS3

Supplement: Figure S3 — A complement to Figure 3. Deletion of Set2 or disruption of Rpd3S causes a general decrease in histone acetylation at promoters. SOM clustering of the enrichment of H4K5ac on promoters of all genes in WT cells along with the difference calculated between the enrichment observed in WT and various mutants. (0.17 MB PDF) [file pgen.1001173.s008.pdf]

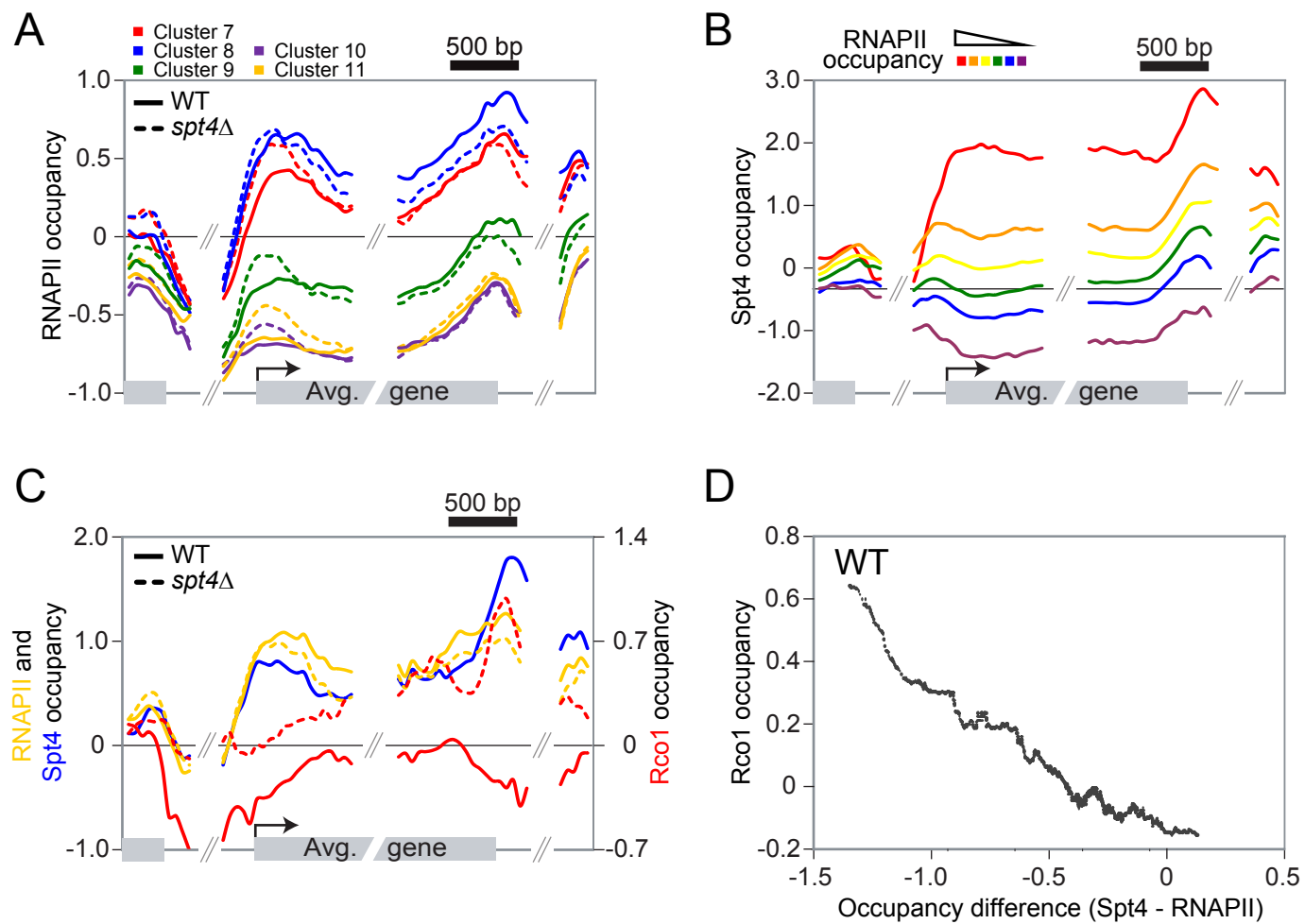

FigS4

Supplement: Figure S4 — A complement to Figure 4. Spt4 negatively regulates the recruitment of Rpd3S. (A) Mapping of RNAPII occupancy in WT (solid lines) and spt4Δ (dashed lines) cells on genes contained within clusters 7 (red, 1136 genes), 8 (blue, 953 genes ), 9 (green, 771 genes ), and the non-identified following clusters 10 (purple, 1031 genes) and 11 (gold, 1109 genes) of the Figure 4. (B) Mapping of Spt4 occupancy on genes binned by their RNAPII occupancy. (C) Mapping of RNAPII (gold), Spt4 (blue) and Rco1 (red) occupancy in WT (solid line) and spt4Δ (dashed line) cells on the 222 genes contained within clusters 4 from Figure 1D. (D) Anti-correlation between Rco1 occupancy and the difference between Spt4 and RNAPII occupancy in WT cells measured on the 2286 transcribed genes (RNAPII>0). A sliding median window of 300 genes was applied to the data. (0.55 MB PDF) [file pgen.1001173.s009.pdf]

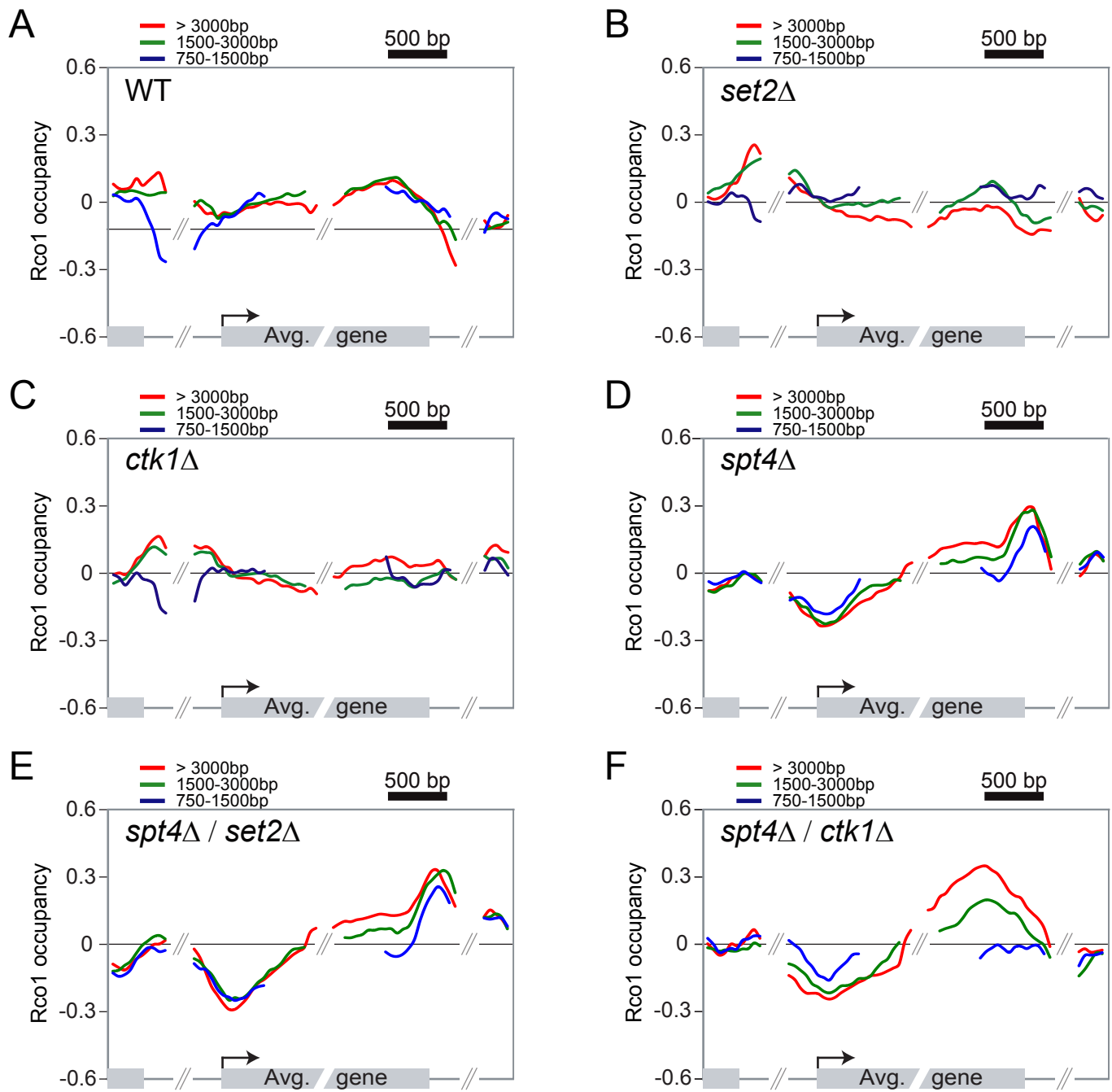

FigS5

Supplement: Figure S5 — Rpd3S ORF occupancy level is gene length-dependant in a spt4Δ/ctk1Δ mutant. (A–D) Mapping of Rco1 occupancy on genes grouped according to their lengths in WT (A), set2Δ (B), ctk1Δ (C), spt4Δ (D), spt4Δ/set2Δ (E), and spt4Δ/ctk1Δ (F) cells (red line: the 465 genes >3000bp, green line: the 1703 genes between 1500–3000bp, blue line: the 2091 genes between 750–1500bp). (0.39 MB PDF) [file pgen.1001173.s010.pdf]
