# Supplementary material for: DSIF and RNA Polymerase II CTD Phosphorylation Coordinate the Recruitment of Rpd3S to Actively Transcribed Genes
Source: PLoS Genet. 2010 Oct 28;6(10):e1001173. doi: 10.1371/journal.pgen.1001173 (PMC2965751; doi:10.1371/journal.pgen.1001173)
Supplement: Table S1 — A list of all the yeast strains used in this study. (0.09 MB DOC) [file pgen.1001173.s011.doc]

| ***Strain*** | ***Alias*** | ***Background*** | ***Genotype*** | ***Source*** |
| --- | --- | --- | --- | --- |
| yFR116 | Wild Type | W303 | *MATa, ade2-1, trp1-1, can1-100, leu2-3,112, his3-11,15, ura3, GAL+, psi+* | (Robert et al., 2004) |
| yFR592 | *eaf3-∆CHD* | W303 | *MATa, ade2-1, trp1-1, can1-100, leu2-3,112, his3-11,15, ura3, GAL+, psi+, eaf3-∆CHD* | This study |
| yFR606 | H3K36A | DY5733 | *MATa,* *trp1-1, ura3-1, his3-11,15, leu2-3,112, ade2-1, can1-100, hht1-hhf1∆::LEU2 hht2-hhf2∆::KanMX3, [hht1(H3K36A)-HHF1 TRP1]* | This study |
| yFR772 | *rco1* | W303 | *MATa, ade2-1, trp1-1, can1-100, leu2-3,112, his3-11,15, ura3, GAL+, psi+, rco1∆::URA3* | This study |
| yFR446 | *rco1-∆PHD* | W303 | *MATa, ade2-1, trp1-1, can1-100, leu2-3,112, his3-11,15, ura3, GAL+, psi+, rco1-∆PHD* | This study |
| yFR248 | *set1; set2; dot1* | W303 | *MATa, ade2-1, trp1-1, can1-100, leu32-3,112, his3-11,15, ura3, GAL+, psi+,dot1∆::HIS3, set2∆::LEU2, set1∆::KanMX4* | (Jin et al., 2007) |
| yFR348 | *set2* | W303 | *MATa, ade2-1, trp1-1, can1-100, leu2-3,112, his3-11,15, ura3, GAL+, psi+, set2∆::HIS3* | This study |
| yFR705 | *spt4* | W303 | *MATa, ade2-1, trp1-1, can1-100, leu2-3,112, his3-11,15, ura3, GAL+, psi+, spt4∆::URA3* | This study |
| yFR847 | *spt4; set2* | W303 | *MATa, ade2-1, trp1-1, can1-100, leu2-3,112, his3-11,15, ura3, GAL+, psi+, spt4∆::URA3, set2∆::LEU2* | This study |
| yFR756 | *ctk1* | W303 | *MATa, ade2-1, trp1-1, can1-100, leu2-3,112, his3-11,15, ura3, GAL+, psi+, spt4∆::URA3, ctk1∆::URA3* | This study |
| yFR891 | *spt5****∆C*** | W303 | *MATa, ade2-1, trp1-1, can1-100, leu2-3,112, his3-11,15, ura3, GAL+, psi+, spt5∆842-1063* | This study |
| yFR763 | *kin28AS* | W303 | *MATα, ade::higG, his3-200, leu2-0, lys2-0, met15-0, trp1-63, ura3-0, kin28-L83G, [pSH579, ARS, CEN, URA3, kin28-L83G]* | This study |
| yFR842 | *bur1AS* | W303 | *MATa, his3-1, leu2-0, met15-0, ura3-0,, bur1-L149G* | This study |
| yFR571 | *RCO1-9myc* | W303 | *MATa, ade2-1, trp1-1, can1-100, leu2-3,112, his3-11,15, ura3, GAL+, psi+, RCO1-9myc-HIS3* | This study |
| yFR630 | *RCO1-9myc eaf3-∆CHD* | W303 | *MATa, ade2-1, trp1-1, can1-100, leu2-3,112, his3-11,15, ura3, GAL+, psi+, RCO1-9myc-HIS3, eaf3-∆CHD* | This study |
| yFR608 | *RCO1-9myc H3K36A* | DY5733 | *MATa, trp1-1, ura3-1, his3-11,15, leu2-3,112, ade2-1, can1-100, hht1-hhf1D::LEU2 hht2-hhf2∆::KanMX3, RCO1-9myc-HIS3, [hht1(H3K36A)-HHF1 TRP1]* | This study |
| yFR636 | *rco1-∆PHD-9myc* | W303 | *MATa, ade2-1, trp1-1, can1-100, leu2-3,112, his3-11,15, ura3, GAL+, psi+, rco1-∆PHD-9myc-HIS3* | This study |
| yFR680 | *RCO1-9myc set1; set2; dot1* | W303 | *MATa, ade2-1, trp1-1, can1-100, leu32-3,112, his3-11,15, ura3, GAL+, psi+,dot1∆::HIS3, set2∆::LEU2, set1∆::KanMX4, RCO1-9myc-TRP1* | This study |
| yFR596 | *RCO1-9myc set2* | W303 | *MATa, ade2-1, trp1-1, can1-100, leu2-3,112, his3-11,15, ura3, GAL+, psi+, RCO1-9myc-HIS3, set2∆::LEU2* | This study |
| yFR706 | *RCO1-9myc spt4* | W303 | *MATa, ade2-1, trp1-1, can1-100, leu2-3,112, his3-11,15, ura3, GAL+, psi+, RCO1-9myc-HIS3, spt4∆::URA3* | This study |
| yFR848 | *RCO1-9myc spt4; set2* | W303 | *MATa, ade2-1, trp1-1, can1-100, leu2-3,112, his3-11,15, ura3, GAL+, psi+, spt4∆::URA3, set2∆::LEU2, RCO1-9myc-HIS3* | This study |
| yFR770 | *RCO1-9myc ctk1* | W303 | *MATa, ade2-1, trp1-1, can1-100, leu2-3,112, his3-11,15, ura3, GAL+, psi+, spt4∆::URA3, ctk1∆::URA3, RCO1-9myc-HIS3* | This study |
| yFR885 | *RCO1-9myc spt4; ctk1* | W303 | *MATa, ade2-1, trp1-1, can1-100, leu2-3,112, his3-11,15, ura3, GAL+, psi+, spt4∆::URA3, ctk1∆::LEU2, RCO1-9myc-HIS3* | This study |
| yFR895 | *RCO1-9myc spt5****∆C*** | W303 | *MATa, ade2-1, trp1-1, can1-100, leu2-3,112, his3-11,15, ura3, GAL+, psi+, spt5∆842-1063, RCO1-9myc-HIS3* | This study |
| yFR868 | *RCO1-9myc KIN28AS* | W303 | *MATα, ade::higG, his3-200, leu2-0, lys2-0, met15-0, trp1-63, ura3-0, RCO1::9myc::HIS3, kin28-L83G, [pSH579, ARS, CEN, URA3, kin28-L83G]* | This study |
| yFR865 | *RCO1-9myc BUR1AS* | W303 | *MATa, his3-1, leu2-0, met15-0, ura3-0,, bur1-L149G, RCO1-9myc-HIS3* | This study |
| yFR170 | *RPD3-18myc* | W303 | *MATa, ade2-1, trp1-1, can1-100, leu2-3,112, ura3, GAL+, psi+, RPD3-18myc-TRP1, his3-11,15* | (Robert et al., 2004) |
| yFR590 | *RXT2-9myc* | W303 | *MATa, ade2-1, trp1-1, can1-100, leu2-3,112, his3-11,15, ura3, GAL+, psi+, RXT2-9myc-TRP1* | This study |
| yFR862 | *SPT4-9myc* | W303 | *MATa, ade2-1, trp1-1, can1-100, leu2-3,112, his3-11,15, ura3, GAL+, psi+, SPT4-9myc-HIS3* | This study |
| yFR588 | *SDS3-9myc* | W303 | *MATa, ade2-1, trp1-1, can1-100, leu2-3,112, his3-11,15, ura3, GAL+, psi+, SDS3-9myc-TRP1* | This study |

References

Jin,Y., Rodriguez,A.M., Stanton,J.D., Kitazono,A.A., and Wyrick,J.J. (2007). Simultaneous mutation of methylated lysine residues in histone H3 causes enhanced gene silencing, cell cycle defects, and cell lethality in Saccharomyces cerevisiae. Mol. Cell Biol. *27*, 6832-6841.

Robert,F., Pokholok,D.K., Hannett,N.M., Rinaldi,N.J., Chandy,M., Rolfe,A., Workman,J.L., Gifford,D.K., and Young,R.A. (2004). Global Position and Recruitment of HATs and HDACs in the Yeast Genome. Mol Cell *16*, 199-209.
