# Supplementary material for: DSIF and RNA Polymerase II CTD Phosphorylation Coordinate the Recruitment of Rpd3S to Actively Transcribed Genes
Source: PLoS Genet. 2010 Oct 28;6(10):e1001173. doi: 10.1371/journal.pgen.1001173 (PMC2965751; doi:10.1371/journal.pgen.1001173)
Supplement: Table S2 — A list of all the microarray hybridizations done in this study. (0.07 MB DOC) [file pgen.1001173.s012.doc]

| ***Experimental Sample*** | | ***Control Sample*** | |
| --- | --- | --- | --- |
| **Antibody** | **Strain** | **Antibody** | **Strain** |
| 8WG16 (RNAPII) | H3K36A | Input DNA | H3K36A |
| 8WG16 (RNAPII) | *rco1* | Input DNA | *rco1* |
| 8WG16 (RNAPII) | *eaf3-∆CHD* | Input DNA | *eaf3-∆CHD* |
| 8WG16 (RNAPII) | *rco1-∆PHD* | Input DNA | *rco1-∆PHD* |
| 8WG16 (RNAPII) | *set1; set2; dot1* | Input DNA | *set1; set2; dot1* |
| 8WG16 (RNAPII) | *set2* | Input DNA | *set2* |
| 8WG16 (RNAPII) | *spt4* | Input DNA | *spt4* |
| 8WG16 (RNAPII) | Wild Type | Input DNA | Wild Type |
| H5 (RNAPII-Ser2P) | Wild Type | Input DNA | Wild Type |
| 9E11 (myc) | *rco1-∆PHD-9myc* | 9E11 (myc) | *rco1-∆PHD* |
| 9E11 (myc) | *RCO1-9myc* | 9E11 (myc) | Wild Type |
| 9E11 (myc) | *RCO1-9myc eaf3-∆CHD* | 9E11 (myc) | *eaf3-∆CHD* |
| 9E11 (myc) | *RCO1-9myc H3K36A* | 9E11 (myc) | H3K36A |
| 9E11 (myc) | *RCO1-9myc set1; set2; dot1* | 9E11 (myc) | *set1; set2; dot1* |
| 9E11 (myc) | *RCO1-9myc set2* | 9E11 (myc) | *set2* |
| 9E11 (myc) | *RCO1-9myc spt4* | 9E11 (myc) | *spt4* |
| 9E11 (myc) | *RCO1-9myc spt4; set2* | 9E11 (myc) | *spt4; set2* |
| 9E11 (myc) | *RCO1-9myc ctk1* | 9E11 (myc) | *ctk1* |
| 9E11 (myc) | *RCO1-9myc spt4; ctk1* | 9E11 (myc) | *ctk1* |
| 9E11 (myc) | *RCO1-9myc spt5****∆C*** | 9E11 (myc) | *spt5****∆C*** |
| 9E11 (myc) | *RCO1-9myc KIN28AS* | 9E11 (myc) | *KIN28AS* |
| 9E11 (myc) | *RCO1-9myc BUR1AS* | 9E11 (myc) | *BUR1AS* |
| 9E11 (myc) | *RPD3-18myc* | 9E11 (myc) | Wild Type |
| 9E11 (myc) | *RXT2-9myc* | 9E11 (myc) | Wild Type |
| 9E11 (myc) | *SDS3-9myc* | 9E11 (myc) | Wild Type |
| 9E11 (myc) | *SPT4-9myc* | 9E11 (myc) | Wild Type |
| H4 | H3K36A | Input DNA | H3K36A |
| H4 | *rco1* | Input DNA | *rco1* |
| H4 | *rco1-∆PHD* | Input DNA | *rco1-∆PHD* |
| H4 | *eaf3-∆CHD* | Input DNA | *eaf3-∆CHD* |
| H4 | *spt4* | Input DNA | *spt4* |
| H4 | *set1; set2; dot1* | Input DNA | *set1; set2; dot1* |
| H4 | Wild Type | Input DNA | Wild Type |
| H4K5Ac | H3K36A | Input DNA | H3K36A |
| H4K5Ac | *rco1* | Input DNA | *rco1* |
| H4K5Ac | *rco1-∆PHD* | Input DNA | *rco1-∆PHD* |
| H4K5Ac | *eaf3-∆CHD* | Input DNA | *eaf3-∆CHD* |
| H4K5Ac | *spt4* | Input DNA | *spt4* |
| H4K5Ac | *set1; set2; dot1* | Input DNA | *set1; set2; dot1* |
| H4K5Ac | *set2* | H4 | *set2* |
| H4K5Ac | Wild Type | Input DNA | Wild Type |
